# Low temperature of radiofrequency ablation at the target sites can facilitate rapid progression of residual hepatic VX2 carcinoma

**DOI:** 10.1186/1479-5876-8-73

**Published:** 2010-07-29

**Authors:** Shan Ke, Xue-mei Ding, Jian Kong, Jun Gao, Shao-hong Wang, Yan Cheng, Wen-bing Sun

**Affiliations:** 1 Department of Hepatobiliary Surgery, West Campus, Beijing Chao-yang Hospital Affiliated to Capital Medical University, Beijing 100043, China

## Abstract

**Background:**

Rapid progression of residual tumor after radiofrequency ablation (RFA) of hepatocellular carcinoma has been observed increasingly. However, its underlying mechanisms remain to be clarified. The present study was designed to determine whether low temperature of RFA at the target sites facilitates rapid progression of residual hepatic VX2 carcinoma and to clarify the possible underlying mechanisms.

**Methods:**

The residual VX2 hepatoma model in rabbits was established by using RFA at 55, 70 and 85°C. Rabbits that were implanted with VX2 hepatoma but did not receive RFA acted as a control group. The relationship between rapid progression of residual hepatic VX2 carcinoma and low temperature of RFA at the target sites was carefully evaluated. A number of potential contributing molecular factors, such as proliferating cell nuclear antigen (PCNA), matrix metalloproteinase 9 (MMP-9), vascular endothelial growth factor (VEGF), hepatocyte growth factor (HGF) and Interleukin-6 (IL-6) were measured.

**Results:**

The focal tumor volume and lung metastases of RFA-treated rabbits increased significantly compared with the control group (*P *< 0.05), and the greatest changes were seen in the 55°C group (*P *< 0.05). Expression of PCNA, MMP-9, VEGF, HGF and IL-6 in tumor tissues increased significantly in the RFA-treated groups compared with the control group, and of the increases were greatest in the 55°C group (*P *< 0.05). These results were consistent with gross pathological observation. Tumor re-inoculation experiments confirmed that low temperature of RFA at the target sites facilitated rapid progression of residual hepatic VX2 carcinoma.

**Conclusions:**

Insufficient RFA that is caused by low temperature at the target sites could be an important cause of rapid progression of residual hepatic VX2 carcinoma. Residual hepatic VX2 carcinoma could facilitate its rapid progression through inducing overexpression of several molecular factors, such as PCNA, MMP-9, VEGF, HGF and IL-6.

## Background

Hepatocellular carcinoma (HCC) is still one of the most important diseases for health care systems due to its high morbidity, mortality and increasing incidence worldwide [1]. Although hepatic resection and transplantation have been considered as the main curative therapies for HCC, the vast majority of patients are not eligible when this tumor is detected. Only about 20% of HCC cases are resectable [2,3]. Currently, various local ablative therapies, such as radiofrequency ablation (RFA), have been accepted as an alternative treatment option for HCC, because of its several advantages, such as definitive therapeutic effect, minimal invasiveness, repeatability, safety, and shorter hospitalization [3].

At present, residual tumor is one of the main obstacles that greatly hinders the effectiveness of RFA for HCC [4]. The residual tumor cannot be entirely avoided for several reasons, such as the mechanisms of RFA, the pathological characteristics of HCC, and the anatomical traits of the liver. The reasons for residual tumor can be categorized as follows: First, the target temperature for ablation cannot be easily reached due to the "heat sink" effect of blood vessels, especially large vessels, within or around the tumor [5]. Second, the operator might deliberately reduce the local intensity of RFA to avoid unintended injury when the tumor is adjacent to an organ such as the stomach, intestine or gallbladder. Third, the performance of overlapping ablation in a mathematically irregular fashion is difficult, especially by the percutaneous route. As a result, nests of viable tumor cells remain in the clefts between the incompletely fused coagulation zones. Finally, the microvascular invasion area that surrounds the main tumor in HCC is sometimes wider than expected, or undetected microscopic satellite tumor lesions might be present [6].

Since 2001, rapid progression of residual tumor after RFA of HCC has been observed increasingly [7,8]. Cumulative evidence has demonstrated that residual tumor after RFA might exhibit an aggressive phenotype and unfavorable prognosis [9], and even change to sarcoma [10], which leads to deterioration of the patient's condition. The conventional concepts of residual tumor have been greatly altered recently. It is believed that clarifying the underlying mechanisms of rapid progression of residual tumor might have a significant effect on the therapeutic principle and strategy of RFA for HCC [8].

Based on analysis of the aforementioned risk factors, we hypothesized that low temperature of RFA at the target sites, which leads to incomplete ablation, might play an important role in facilitating rapid progression of residual tumor of HCC after RFA. The present study was designed to test this hypothesis and to clarify the possible underlying mechanisms.

## Methods

### Animals and tumor inoculation

**Figure 1:**
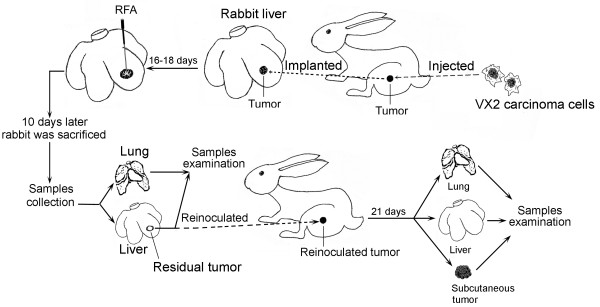
**Schematic diagram of the whole experimental plan**.

The experiments were performed with New Zealand white rabbits that weighed 2.5-3.0 kg. The experiments were approved by the Animal Care Committee of Capital Medical University, Beijing, China and were performed in accordance with the institutional guidelines. The animals were anesthetized with an intravenous injection of 35 mg/kg pentobarbital. The animals were allowed food and water *ad libitum *between the various procedures. A schematic diagram has been produced and added to illustrate the experimental procedures (Fig. 1). VX2 carcinoma was used to establish the model of HCC. VX2 carcinoma is an anaplastic squamous cell carcinoma that is derived from a virus-induced papilloma in wild rabbits, but appears as a carcinoma in the domestic species. VX2 tumors were first grown for 2 weeks on the hind legs of carrier rabbits and then were harvested after they reached a size of 1.5 cm. The harvested tumors were placed into saline solution and cut into cubes of 1 mm^3^. Only portions of tumor tissue that did not show any macroscopic signs of necrosis were used. Abdomens of the recipient rabbits were shaved and prepared with povidone iodine, and a midline subxyphoid incision was made. The anterior surface of the liver was exposed and one of the prepared cubes of tumor tissue was implanted in the left lobe of the liver using a 21-gauge angioneedle (Becton Dickinson, Sandy, UT, USA). This method allowed the growth of a solitary, well-demarcated tumor. There was only one inoculation site in each liver. Proper aseptic technique was rigorously observed during each inoculation. After surgery, the animals were returned to their cages, kept warm, and monitored in the animal laboratory until they recovered from anesthesia. An HDI 5000 ultrasound system (Philips Healthcare, Bothell, WA, USA) with a 7.5-MHz linear probe was used to monitor the tumor size. Based on the methods described previously[11,12] and our experimental design, VX2 carcinoma nodules > 2.0 cm in diameter were considered appropriate for RFA. The period for tumors to reach the size of 2.0 cm ranged from 16 to 18 days. All inoculations were performed by the same individual investigator, who inoculated specimens of the same tumor into all rabbits to minimize inter-animal variations in tumor growth rate.

### Model of residual hepatic VX2 carcinoma following RFA

**Figure 2:**
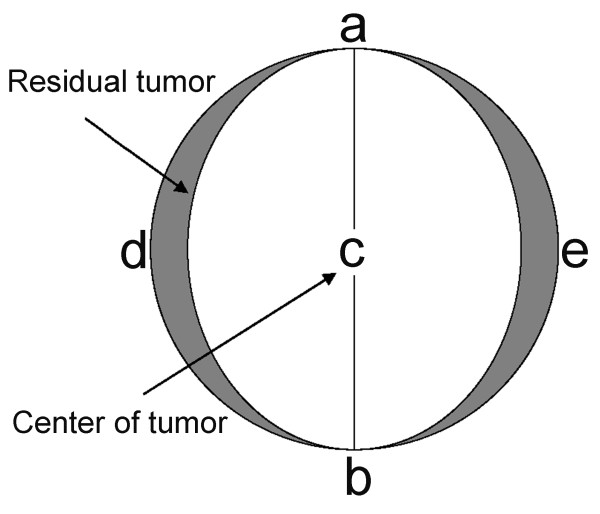
**Sketch of residual hepatic VX2 carcinoma following RFA**. The oval-shaped area of "adbe" represents the whole tumor. The line "ab" is the minor axis of the tumor and the line "de" is the major axis. The tumor center was also designated as the RFA center. The measured minor axis of the tumor was used to guide the release of the RFA needle electrode. Thus, residual tumor was left on both sides of the measured major tumor axis (shadowed area).

The RFA procedure for residual hepatic VX2 carcinoma was standardized in advance as depicted in Fig. 1 and Fig. 2. Sixty rabbits were randomly divided into four groups of 15: group I was treated with RFA at 55°C; group II was treated with RFA at 70°C; group III was treated with RFA at 85°C; and the control group received laparotomy, RFA probe puncture but no ablation. RFA was performed using the same anesthesia protocol as for carcinoma implantation. Two grounding pads were applied to the animal's flank before RFA. Abdomens of the experimental rabbits were shaved and prepared with povidone iodine, and a midline subxyphoid incision was made. The tumor size was measured and the tumor was ablated. The tumor center was also designated as the RFA center. The measured minor axis of the tumor was used to guide the release of the RFA needle electrode. Thus, residual tumor was left on both sides of the measured major axis of the tumor (shadowed area depicted in Fig. 2). An RF current generator (Model 1500X Generator; RITA Medical Systems, Manchester, GA, USA) was used to generate RF energy. To deliver RF energy, we used a 14-gauge expandable RF needle electrode (StarBurst™ XL; RITA Medical Systems), 10 cm in length. Each ablation cycle lasted for 5 min.

### Gross pathological analysis

The rabbits were sacrificed by injecting an overdose of ketamine and xylazine 10 days after RFA. The liver and lungs were carefully dissected and excised. The number and size of masses were noted. The pathological findings could thus be compared directly with the tumor numbers, volumes and locations. Quantitative evaluation of the lung metastatic nodules was made by two observers using the following procedures: macroscopic study by stereoscopic magnifying glass (Olympus SZH, Zeiss Stemi DV4, Germany) and counting the metastatic nodules on the pleural surface of the whole lobules [13]. The variation between the observers' findings was < 5%.

### Immunohistochemical analysis

The streptavidin-peroxidase two-step method was used for immunohistochemical detection of matrix metalloproteinase 9 (MMP-9), vascular endothelial growth factor (VEGF) and proliferating cell nuclear antigen (PCNA). Representative 5-μm tissue sections were cut from paraffin-embedded specimens. The sections were washed three times for 3 min with PBS, and blocked with a solution of 30 mL/L hydrogen peroxide in ethanol for 10 min at room temperature. They were immersed in 30 mL/L normal horse serum for 10 min at room temperature. The sections were incubated for 1 h with primary antibodies (mouse monoclonal antibodies; Abcam, Cambridge, UK) specific to MMP-9 (dilution 1:50), VEGF (dilution 1:50) or PCNA (dilution 1:100). Negative controls consisted of tissue sections incubated with Tris-buffered saline (TBS) instead of the primary antibody. The immunoreactivity was then visualized by incubating the samples in 3,3'-diaminobenzedine. Finally, the slides were counterstained with hematoxylin. To evaluate the expression of MMP-9, VEGF and PCNA, all slides were examined and scored by two independent pathologists who were blinded to the animal data. A few cases with discrepant scores were reevaluated to reach a final agreement. Any slides that exhibited diffuse immunostaining or > 50% tumor cells were classified as (++), > 10% but < 50% as (+), and < 10% as (-).

### Western blotting

Proteins for Western blotting were isolated from fresh-frozen tissue using T-Per extraction reagent (Pierce Biotechnology, Rockford, IL, USA) according to the manufacturer's recommendations. The supernatants were frozen at -80°C until use. The proteins were fractionated by 10% SDS-PAGE and followed by electrotransfer onto nitrocellulose filters (Bio-Rad, CA, USA). The filters were blocked at 4°C overnight with a blocking buffer (pH 7.6) that contained 5% non-fat dry milk. The filters were incubated with a primary monoclonal antibody to MMP-9 (1:200; Abcam), VEGF (1:200; Abcam), PCNA (1:200; Abcam), and a secondary antimouse HRP-antibody (1:2000; Santa Cruz Biotechnology, Santa Cruz, CA, USA) for 2 h at room temperature. Immunoreactive bands were visualized using ECL detection reagents (Amersham Pharmacia Biotech, Little Chalfont, Bucks, UK).

### ELISA

Expression of hepatocyte growth factor (HGF) in tissues was measured using the ELISA Kit for Rabbit HGF according to the manufacturer's instructions (USCN Life Science, Wuhan, China). Expression of interleukin 6 (IL-6) in tissues was measured using the ELISA Kit for Rabbit IL-6 according to the manufacturer's instructions (USCN Life Science, Wuhan, China).

### Tumor reinoculation

The rabbits were sacrificed and the liver tumors were harvested simultaneously 10 days after RFA. The harvested tumors were placed in saline solution and cut into cubes of 1 mm^3^. Only portions of tumor tissue that did not show any macroscopic signs of necrosis were used. The tumor tissue was reinoculated subcutaneously into the hind legs of the rabbits as depicted in Fig. 1. Tumor sizes were measured every 1-2 days, with tumor volumes calculated according to the formula [14]: V = ab^2^/2, where, a is the longest and b the smallest diameter of the tumor *in vivo*. Rabbits were sacrificed 21 days after reinoculation or when they became moribund. The tumor, liver and lungs were carefully dissected and examined.

### Statistical analysis

Data were presented as the means ± SD for the indicated number of separate experiments. Statistical analysis was performed using SPSS version 11.5. One-way ANOVA followed by the Newman-Keuls test, Kruskal-Wallis H test, Mann-Whitney test and Student's *t *test were used to evaluate statistical significance and *P *< 0.05 was considered significant.

## Results

### Effects of low temperature of RFA at the target sites on growth of hepatic VX2 carcinoma

**Figure 3:**
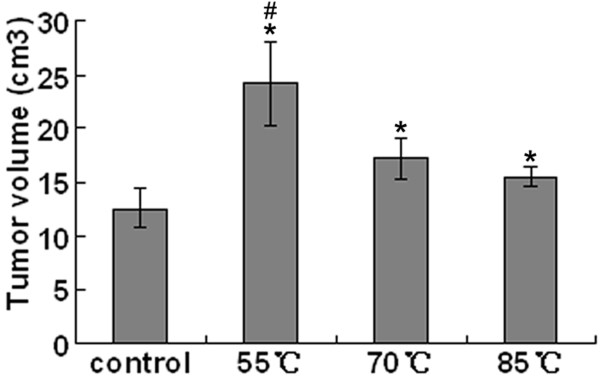
**Growth of hepatic VX2 carcinoma after insufficient RFA due to low temperature at the target sites**. Data were expressed as means ± SD of three independent experiments. (*P < 0.05, groups I, II and III *vs*. control group. #*P *< 0.05, group I *vs*. groups II and III, by one-way ANOVA and Newman-Keuls test).

We showed in our previous experiments that residual hepatic VX2 carcinoma could be seen microscopically in groups I, II and III. We determined the effects of 5 min RFA at each temperature on the growth of hepatic VX2 carcinoma. The changes in tumor volume were 24.21 ± 3.94 cm^3 ^in group I, 17.28 ± 1.84 cm^3 ^in group II and 15.48 ± 0.91 cm^3 ^in group III, which were all larger than that in the control group (12.63 ± 1.87 cm^3^). It seemed that the lower the temperature of RFA was, the larger the tumor volume was (Fig. 3).

### Effects of low temperature of RFA at the target sites on lung metastasis of hepatic VX2 carcinoma

**Figure 4:**
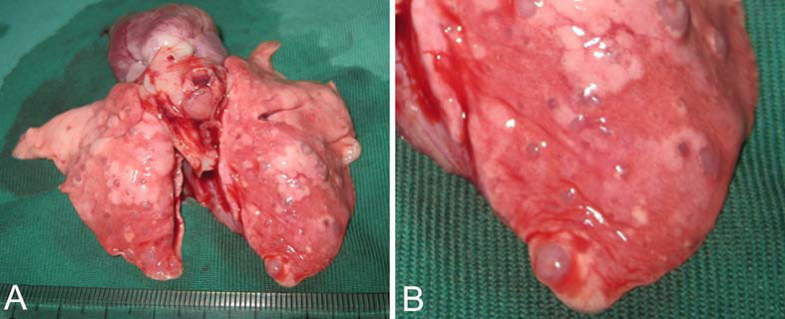
**Macroscopic characteristics of pulmonary metastatic nodules**. A. Macroscopic view of the lung. B. Fractionated view of the lung, which has been magnified to show the details of the metastatic nodules.

**Figure 5:**
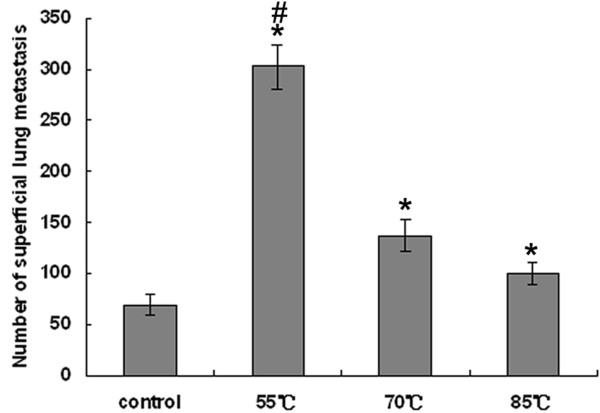
**Frequency of pulmonary metastatic nodules in the control group and groups treated with RFA at different low target temperatures**. Data were expressed as means ± SD of three independent experiments. (*P < 0.05, groups I, II and III *vs*. control group. #*P *< 0.05, group I *vs*. groups II and III by one-way ANOVA and Newman-Keuls test).

We examined by gross observation lung metastasis of hepatic VX2 carcinoma after RFA at low temperature. Quantifiable metastatic nodules were taken to be those structures of a white-grey coloration that could be distinguished on the lung surface and which was sufficiently separated from each other to be counted individually (Fig. 4A and 4B). The numbers of metastatic nodules are shown in Fig. 5. The control group had between 55 and 80 metastatic nodules randomly distributed over the lung surface, with a mean of 69.0 ± 10.5. Group I showed a mean of 302.2 ± 21.6, group II, a mean of 137.2 ± 16.3, and group III, a mean of 99.6 ± 10.5. Group I was treated with the lowest temperature, therefore, it showed the greatest increase in metastatic nodules compared with all the other groups (*P *< 0.05).

### Immunohistochemical assay

**Figure 6:**
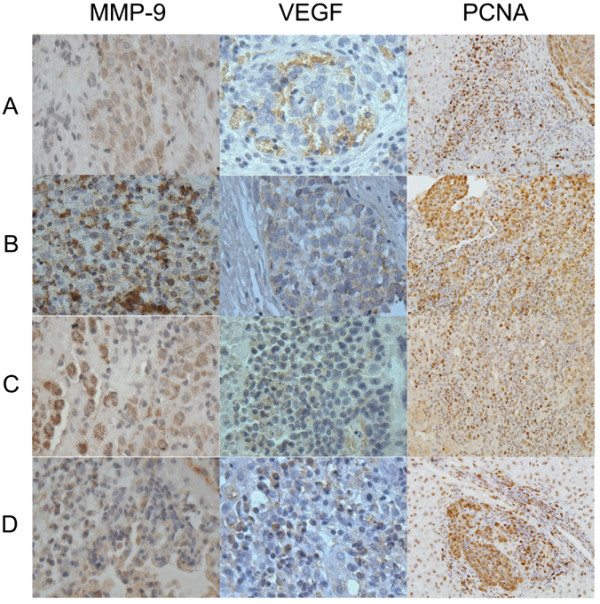
**Immunohistochemical staining for MMP-9, VEGF and PCNA in residual hepatic VX2 carcinoma tissues**. Original magnifications: MMP-9 and VEGF, ×400; PCNA, ×200. A, control group; B, group I; C, group II; D, group III.

MMP-9, VEGF and PCNA were found to be mainly expressed in cancerous lesions, but also in some normal tissues (Fig. 6). *In vitro *cell invasiveness was assessed using anti-MMP-9 antibody. *In vitro *cell proliferation and angiogenesis were evaluated using anti-PCNA and anti-VEGF antibodies, respectively (Fig. 6). The percentage of positive MMP-9, VEGF and PCNA tumor cells in the RFA treatment groups were markedly higher than that in the control group (Table 1, *P *< 0.05). Compared with groups II and III, the percentage of positive MMP-9, VEGF and PCNA tumor cells in group I was even higher (*P *< 0.05).

**Table 1:** Immunohistochemical results of MMP-9, VEGF and PCNA

	Group	N	Expression density	No of cases	%	*P*
MMP-9	I	15				
			++	13	86.67	< 0.01
			+	2	13.33	
			-	0	0.00	
	II	15				
			++	5	33.33	
			+	9	60.00	
			-	1	6.67	
	III	15				
			++	4	26.67	
			+	10	66.67	
			-	1	6.67	
	Control	15				
			++	0	0.00	
			+	7	46.67	
			-	8	53.33	
VEGF	I	15				
			++	12	80.00	< 0.01
			+	3	20.00	
			-	0	0.00	
	II	15				
			++	5	33.33	
			+	9	60.00	
			-	1	6.67	
	III	15				
			++	3	20.00	
			+	10	66.67	
			-	2	13.33	
	Control	15				
			++	0	0.00	
			+	6	40.00	
			-	9	60.00	
PCNA	I	15				
			++	11	73.33	< 0.01
			+	4	26.67	
			-	0	0.00	
	II	15				
			++	4	26.67	
			+	10	66.67	
			-	1	6.67	
	III	15				
			++	2	13.33	
			+	12	80.00	
			-	1	6.67	
	Control	15				
			++	0	0.00	
			+	5	33.33	
			-	10	66.67	

MMP-9: matrix metalloproteinase 9; VEGF: vascular endothelial growth factor; PCNA: proliferating cell nuclear antigen. Any slides that exhibited diffuse immunostaining or > 50% tumor cells were classified as (++), > 10% but < 50% as (+), and < 10% as (-). (*P *< 0.01, by Kruskal-Wallis H test)

### MMP-9, VEGF and PCNA expression in residual hepatic VX2 carcinoma tissues

**Figure 7:**
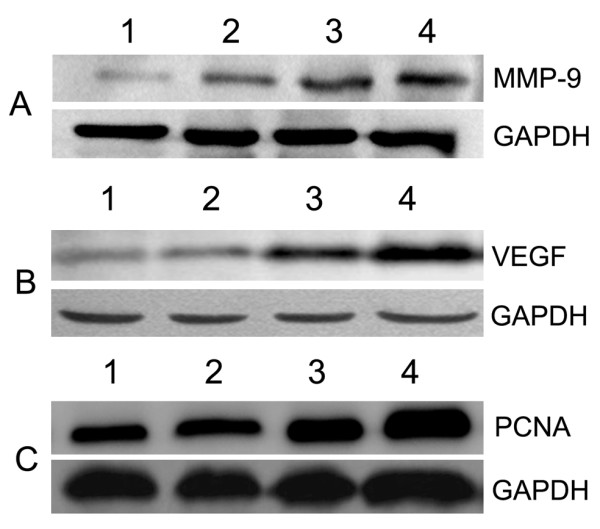
**MMP-9, VEGF and PCNA expression in residual hepatic VX2 carcinoma tissues**. A, MMP-9; B, VEGF; C, PCNA. 1, control group; 2, group III; 3, group II; 4, group I.

Expression of MMP-9 in tumor tissues was markedly decreased in the control group, and incomplete RFA due to low temperature at the target sites significantly increased MMP-9 level in the other groups (Fig. 7). Similarly, incomplete RFA significantly elevated protein expression of VEGF and PCNA in groups I, II and III. At the same time, expression of VEGF and PCNA was markedly decreased in tumor tissues in the control group (Fig. 7). The lower the target temperature of RFA was, the higher was the expression of MMP-9, VEGF and PCNA in residual hepatic VX2 carcinoma tissues. These results had a similar trend as those of the immunohistochemical assays.

### Tissue levels of HGF and IL-6 at the time of animal killing

**Figure 8:**
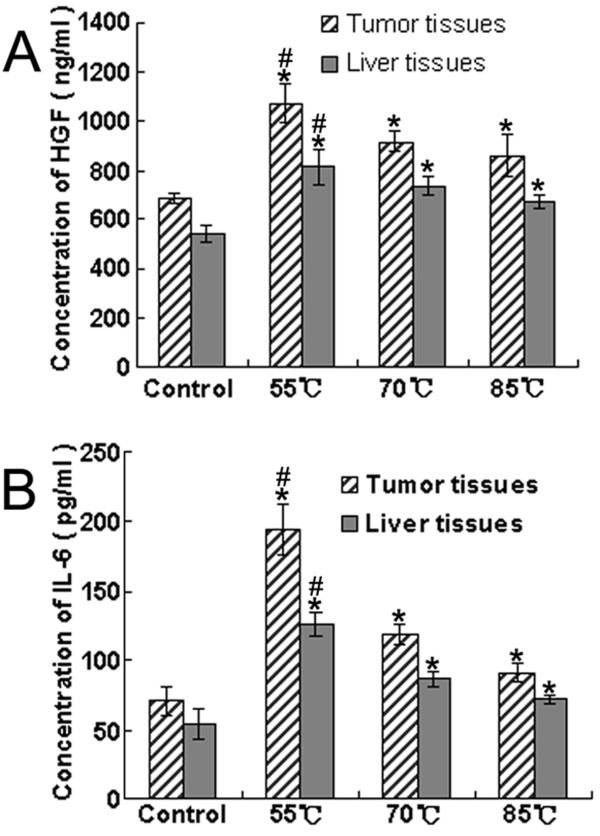
**RFA might affect the expression of HGF and IL-6 in liver and tumor tissues**. A, Concentration of HGF; B, Concentration of IL-6. Liver tissues represented the normal tissues from the same liver which was treated with RFA. Data were expressed as means ± SD of three independent experiments. (*P < 0.05, groups I, II and III *vs*. control group. #*P *< 0.05, group I *vs*. groups II and III, by one-way ANOVA and Newman-Keuls test).

To assess whether any cytokines from the tumor microenvironment might be involved in rapid tumor progression after incomplete RFA, the levels of HGF and IL-6 were determined in tumor and normal tissues from the same liver at the time of animal killing. Expression of HGF and IL-6 in the tumor tissues of group I increased dramatically in comparison with that in the control group. At the same time, the concentrations of HGF and IL-6 in non-ablated liver tissues in group I were much higher than those in liver tissues in the control group (Fig. 8).

### Measurement of tumor growth and metastasis after reinoculation

**Figure 9:**
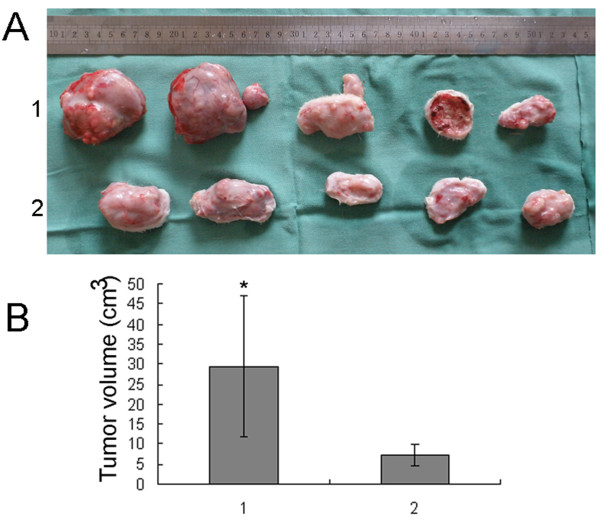
**Volumes of hepatic VX2 carcinoma 21 days after reinoculation**. A, Photographs of the hepatic VX2 carcinoma of five rabbits selected from the control group and five from group I. 1, Group I, RFA at 55°C; 2, control group. The top row shows hepatic VX2 carcinoma of rabbits treated with RFA, and large tumors were seen. The bottom row shows hepatic VX2 carcinoma of control rabbits with smaller tumors. B, 1, group I, RFA at 55°C; 2, control group. Data are expressed as means ± SD of three independent experiments. (*P < 0.05, by Student's *t *test)

**Figure 10:**
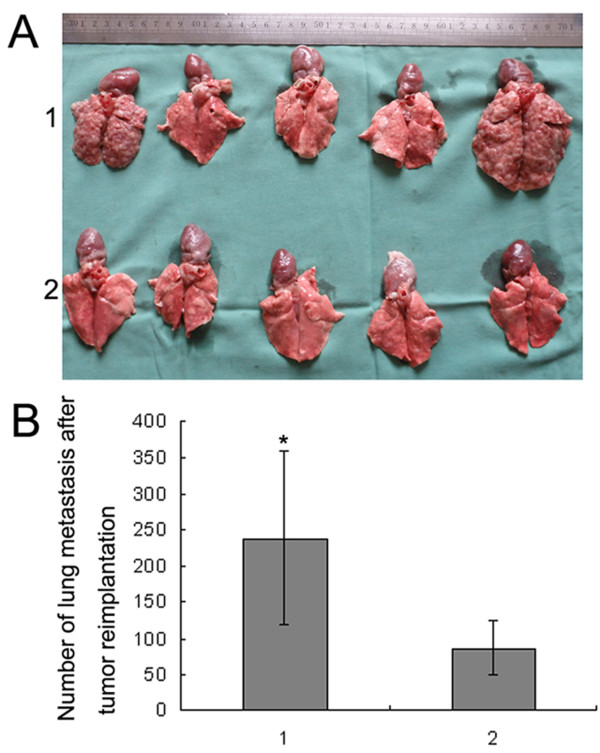
**Lung metastasis of hepatic VX2 carcinoma 21 days after reinoculation**. A, Photographs of the lungs of five rabbits selected from the control group and five from group I. 1, group I, RFA at 55°C; 2, control group. The top row shows lungs of rabbits treated with RFA, and numerous, large, white-grey tumors were seen. The bottom row shows lungs of control rabbits with fewer and smaller tumors. B, 1, group I, RFA at 55°C; 2, control group. Data are expressed as means ± SD of three independent experiments. (*P < 0.05, by Student's *t *test)

To demonstrate further the role of low temperature of RFA at the target sites in the rapid progression of residual hepatic VX2 carcinoma, tumor tissues that survived the first RFA at 55°C were reinoculated to other normal rabbits. The tumor growth and metastasis were carefully observed individually. It was found that tumor growth and lung metastasis in the RFA groups were much more obvious than in the control group (Figs. 9 and 10). However, no liver metastasis was detected in any of the groups.

## Discussion

It was demonstrated directly or indirectly in our study that residual tumor was prone to proliferation, invasion and metastasis when the local ablative temperature was not sufficiently high. Besides, it seemed that the lower the target temperature was, the more significant were the local proliferation and distant metastasis (e.g. to the lungs) of the tumor. It is known that different cells, such as tumor cells, have different endurance to heat. Some cells can even survive high temperature from 50-55°C. However, cells can seldom survive temperatures above 55°C [9]. Therefore, it seems that most residual tumor and rapid progression should occur when the temperature is below 55°C, which is consistent with the results of our study.

In clinical settings, although the target temperature can be set as high as 105-115°C during RFA, only the tissues that surround the electrodes can reach that temperature [15]. In fact, the real temperature of the tumor tissue between the two adjacent electrodes is lower than the target temperature because of the "heat sink" effect of blood flow [5]. Residual tumor can occur whenever the local ablative temperature is sufficiently low. In the present study, the VX2 nodules were transplanted into the liver rather than derived from the liver itself, therefore, the feeding artery and the heat sink effect were less than those for HCC. The real ablative temperature of the tumor might be nearer to the target temperature, compared with the clinical situation. This was why we chose 85°C as the highest RFA temperature.

At present, a large number of molecular factors have been shown to be associated with HCC invasion and metastasis, such as PCNA, MMP-9, VEGF, HGF and IL-6. PCNA is a nuclear protein that plays a key role in cell proliferation, DNA repair and cell cycle control [16]. In cirrhotic patients, a high level of PCNA immunolabeling is associated with an increased risk of HCC [17,18], and in HCC, high PCNA values are associated with poor prognosis [17]. Cell invasion is a major component of the complex multistep process of tumor metastasis. Invasion of malignant tumor cells requires destruction of basement membranes and proteolysis of extracellular matrix (ECM). Of the several families of ECM-degrading enzymes, the most extensive are the MMPs, which are a large family of structurally related zinc endopeptidases that collectively degrade most of the ECM components [19,20]. Among previously reported human MMPs, MMP-9 is thought to be a vital enzyme for degrading type IV collagen and is postulated to play an important role in HCC invasion and metastasis [21,22].

Tumor angiogenesis is another crucial step in the growth and metastasis of cancer, including HCC, and has drawn much attention in recent years [23,24]. Hence, the molecular basis of tumor angiogenesis has been a major interest in the field of cancer research. The VEGF pathway is well established as an important driving force of this process [25]. To date, increasing evidence indicates that tumor-stromal cell interactions have a crucial role in tumor initiation and progression [26]. These interactions modify cellular compartments, which leads to the co-evolution of tumor cells and their microenvironment. HGF, also known as scatter factor, is produced by non-parenchymal liver cells, and is a multifunctional cytokine of the tumor microenvironment of HCC [27]. HGF accomplishes most of the functions of the invasive program in carcinomas (loss of adhesive junctions, motility, angiogenesis, and survival/apoptosis). HGF receptor, also known as c-Met, plays important roles in angiogenesis and tumor growth [28]. It has been reported that c-Met expression is significantly higher in the invasive type of HCC, as determined by gross type, vessel invasion, intrahepatic metastasis and histological type, and induction of c-Met might be used as a indicator of HCC progression, especially intrahepatic metastasis [29].

IL-6 is a multifunctional regulator of the immune response and hematopoiesis. Recently, it has been reported that expression of IL-6 is correlated with prognosis in various cancer patients [30-32]. Kanazawa et al. [32] have reported that IL-6 can directly influence cell proliferation and the invasion potential as the first step of tumor metastasis. Hong et al. [33] believe that therapeutic targeting of IL-6 and its receptor in cancer has a strong biological rationale, and there is preliminary evidence to suggest that targeting of the IL-6 system might be beneficial in the treatment of cancer. In the present study, it was shown that expression of PCNA, MMP-9, VEGF, HGF and IL-6 in tumor tissues in groups I, II and III, which received incomplete RFA, increased remarkably. Furthermore, it seemed that the lower the target temperature of RFA was, the higher was the expression of these molecular factors. This was consistent with the results of tumor inoculation and reinoculation studies. These data suggest that the residual tumor cells facilitate tumor growth and metastasis through induction of overexpression of PCNA, MMP-9, VEGF, HGF and IL-6.

It is important to clarify the underlying mechanisms of rapid progression of residual HCC after RFA, to optimize the therapeutic principles and strategies of RFA. It has been reported that HGF/c-Met signaling can activate multiple signal transduction pathways, including the Src/focal adhesion kinase pathway, the p120/signal transducer and activator of transcription 3 pathway, the phosphoinositide-3 kinase (PI3K)/Akt pathway, and the MEK/ERK pathway [34,35]. It has been confirmed that the PI3K/Akt and MEK/ERK pathways play a vital role in tumor invasion and metastasis [36-40]. Increasing evidence has demonstrated that the HGF/c-Met signaling pathway could be another valuable pathway for research on tumor target therapy, besides the VEGF signal pathway. HGF/c-Met signaling is activated in angiogenesis and tumor growth, therefore, several strategies have been explored for inhibiting this pathway. Some inhibitors of the HGF/c-Met signaling pathway have been developed and introduced into preclinical and phase I and II clinical trials [41]. In our study, we found that expression of HGF in tumor tissues after incomplete RFA was much higher than that in tumor tissues without RFA. This indicates that the HGF/c-Met signaling pathway might be involved in the rapid progression of residual tumor after RFA. Further research in this area could have potential for enhancing the therapeutic effect of RFA on HCC. Another significant finding was that expression of HGF and IL-6 in non-ablated liver tissues in group I were much higher than those in liver tissues in the control group. We supposed that both the liver injury triggered by RFA and autocrine loop in tumor cells may involve in it. However, this needs to be confirmed.

## Conclusions

In conclusion, the results of this study highlight two issues. Firstly, insufficient RFA, which is caused by low temperature at the target sites, could be an important reason for rapid progression of residual hepatic VX2 carcinoma. Secondly, residual hepatic VX2 carcinoma might facilitate rapid tumor progression through induction of overexpression of multiple molecular factors, such as PCNA, MMP-9, VEGF, HGF and IL-6.

## Competing interests

The authors declare that they have no competing interests.

## Authors' contributions

SK, XD and JK performed the rabbit experiments and ELISA analysis. JG, SW and YC carried out the immunohistochemistry, XD and JK performed the western blotting. SK, XD and WS conceived and designed the experiments and analyzed the data. The manuscript was written by SK and WS. All authors read and approved the final manuscript.

## References

[B1] Shariff MI Cox IJ Gomaa AI Khan SA Gedroyc W Taylor-Robinson SD Hepatocellular carcinoma: current trends in worldwide epidemiology, risk factors, diagnosis and therapeutics Expert Rev Gastroenterol Hepatol 2009 3 4 353 367 10.1586/egh.09.35 19673623

[B2] Sun WB How is radiofrequency ablation going in treating hepatocellular carcinoma in China? Austral-Asian Journal of Cancer 2008 7 4 221 224

[B3] Lau WY Lai EC The current role of radiofrequency ablation in the management of hepatocellular carcinoma: a systematic review Ann Surg 2009 249 1 20 25 10.1097/SLA.0b013e31818eec29 19106671

[B4] Rhim H Kim YS Choi D Lim HK Park K Percutaneous radiofrequency ablation of hepatocellular carcinoma: analysis of 80 patients treated with two consecutive sessions Eur Radiol 2008 18 7 1442 1448 10.1007/s00330-008-0902-4 18389252

[B5] Thanos L Mylona S Galani P Pomoni M Pomoni A Koskinas I Overcoming the heat-sink phenomenon: successful radiofrequency thermal ablation of liver tumors in contact with blood vessels Diagn Interv Radiol 2008 14 1 51 56 18306146

[B6] Zhou XP Yang GS Cong WM Lu JH Zhang SH Zong M Retrospective and prospective study on micrometastasis in liver parenchyma surrounding PLC Chinese Journal of Hepatobiliary Surgery 2005 11 8 510 514

[B7] Seki T Tamai T Ikeda K Imamura M Nishimura A Yamashiki N Nakagawa T Inoue K Rapid progression of hepatocellular carcinoma after transcatheter arterial chemoembolization and percutaneous radiofrequency ablation in the primary tumour region Eur J Gastroenterol Hepatol 2001 13 3 291 294 10.1097/00042737-200103000-00014 11293452

[B8] Ruzzenente A Manzoni GD Molfetta M Pachera S Genco B Donataccio M Guglielmi A Rapid progression of hepatocellular carcinoma after Radiofrequency Ablation World J Gastroenterol 2004 10 8 1137 1140 15069713 10.3748/wjg.v10.i8.1137 PMC4656348

[B9] Obara K Matsumoto N Okamoto M Kobayashi M Ikeda H Takahashi H Katakura Y Matsunaga K Ishii T Okuse C Suzuki M Itoh F Insufficient radiofrequency ablation therapy may induce further malignant transformation of hepatocellular carcinoma Hepatol Int 2008 2 1 116 123 10.1007/s12072-007-9040-3 19669287 PMC2716878

[B10] Kasugai H Osaki Y Oka H Kudo M Seki T Severe complications of radiofrequency ablation therapy for hepatocellular carcinoma: an analysis of 3,891 ablations in 2,614 patients Oncology 2007 72 Suppl 1 72 75 10.1159/000111710 18087185

[B11] Kim TJ Moon WK Cha JH Goo JM Lee KH Kim KH Lee JW Han JG Weinmann HJ Chang KH VX2 carcinoma in rabbits after radiofrequency ablation: comparison of MR contrast agents for help in differentiating benign periablational enhancement from residual tumor Radiology 2005 234 2 423 430 10.1148/radiol.2342031456 15591437

[B12] Gu T Li CX Feng Y Wang Q Li CH Li CF Trans-arterial gene therapy for hepatocellular carcinoma in a rabbit model World J Gastroenterol 2007 13 14 2113 2117 17465458 10.3748/wjg.v13.i14.2113 PMC4319135

[B13] Martinez C Vicente V Yanez MJ Garcia JM Canteras M Alcaraz M Experimental model of pulmonary metastasis treatment with IFNalpha Cancer Lett 2005 225 1 75 83 10.1016/j.canlet.2004.11.047 15922859

[B14] Carlsson G Gullberg B Hafstrom L Estimation of liver tumor volume using different formulas - an experimental study in rats J Cancer Res Clin Oncol 1983 105 1 20 23 10.1007/BF00391826 6833336 PMC12252809

[B15] Rossi S Garbagnati F Lencioni R Allgaier HP Marchiano A Fornari F Quaretti P Tolla GD Ambrosi C Mazzaferro V Blum HE Bartolozzi C Percutaneous radio-frequency thermal ablation of nonresectable hepatocellular carcinoma after occlusion of tumor blood supply Radiology 2000 217 1 119 126 11012432 10.1148/radiology.217.1.r00se02119

[B16] Stoimenov I Helleday T PCNA on the crossroad of cancer Biochem Soc Trans 2009 37 Pt 3 605 613 10.1042/BST0370605 19442257

[B17] Stroescu C Dragnea A Ivanov B Pechianu C Herlea V Sgarbura O Popescu A Popescu I Expression of p53, Bcl-2, VEGF, Ki67 and PCNA and prognostic significance in hepatocellular carcinoma J Gastrointestin Liver Dis 2008 17 4 411 417 19104702

[B18] Ballardini G Groff P Zoli M Bianchi G Giostra F Francesconi R Lenzi M Zauli D Cassani F Bianchi F Increased risk of hepatocellular carcinoma development in patients with cirrhosis and with high hepatocellular proliferation J Hepatol 1994 20 2 218 222 10.1016/S0168-8278(05)80061-3 7911817

[B19] Roy R Yang J Moses MA Matrix metalloproteinases as novel biomarkers and potential therapeutic targets in human cancer J Clin Oncol 2009 27 31 5287 5297 10.1200/JCO.2009.23.5556 19738110 PMC2773480

[B20] Li X Wu JF Recent developments in patent anti-cancer agents targeting the matrix metalloproteinases (MMPs) Recent Pat Anticancer Drug Discov 5 2 109 141 10.2174/157489210790936234 19951249

[B21] Yang P Yuan W He J Wang J Yu L Jin X Hu Y Liao M Chen Z Zhang Y Overexpression of EphA2, MMP-9, and MVD-CD34 in hepatocellular carcinoma: Implications for tumor progression and prognosis Hepatol Res 2009 39 12 1169 1177 10.1111/j.1872-034X.2009.00563.x 19788698

[B22] Chen JS Wang Q Fu XH Huang XH Chen XL Cao LQ Chen LZ Tan HX Li W Bi J Zhang LJ Involvement of PI3K/PTEN/AKT/mTOR pathway in invasion and metastasis in hepatocellular carcinoma: Association with MMP-9 Hepatol Res 2009 39 2 177 186 10.1111/j.1872-034X.2008.00449.x 19208038

[B23] Raskopf E Vogt A Sauerbruch T Schmitz V siRNA targeting VEGF inhibits hepatocellular carcinoma growth and tumor angiogenesis in vivo J Hepatol 2008 49 6 977 984 10.1016/j.jhep.2008.07.022 18845354

[B24] Sugimachi K Tanaka S Terashi T Taguchi K Rikimaru T Sugimachi K The mechanisms of angiogenesis in hepatocellular carcinoma: angiogenic switch during tumor progression Surgery 2002 131 1 Suppl S135 141 10.1067/msy.2002.119365 11821800

[B25] Hicklin DJ Ellis LM Role of the vascular endothelial growth factor pathway in tumor growth and angiogenesis J Clin Oncol 2005 23 5 1011 1027 10.1200/JCO.2005.06.081 15585754

[B26] Polyak K Haviv I Campbell IG Co-evolution of tumor cells and their microenvironment Trends Genet 2009 25 1 30 38 10.1016/j.tig.2008.10.012 19054589

[B27] Desiderio MA Hepatocyte growth factor in invasive growth of carcinomas Cell Mol Life Sci 2007 64 11 1341 1354 10.1007/s00018-007-7050-x 17415522 PMC11136139

[B28] You WK McDonald DM The hepatocyte growth factor/c-Met signaling pathway as a therapeutic target to inhibit angiogenesis BMB Rep 2008 41 12 833 839 19123972 10.5483/bmbrep.2008.41.12.833 PMC4417610

[B29] Osada S Kanematsu M Imai H Goshima S Clinical significance of serum HGF and c-Met expression in tumor tissue for evaluation of properties and treatment of hepatocellular carcinoma Hepatogastroenterology 2008 55 82-83 544 549 18613405

[B30] Hodge DR Hurt EM Farrar WL The role of IL-6 and STAT3 in inflammation and cancer Eur J Cancer 2005 41 16 2502 2512 10.1016/j.ejca.2005.08.016 16199153

[B31] Paule B Terry S Kheuang L Soyeux P Vacherot F de la Taille A The NF-kappaB/IL-6 pathway in metastatic androgen-independent prostate cancer: new therapeutic approaches? World J Urol 2007 25 5 477 489 10.1007/s00345-007-0175-6 17541600

[B32] Kanazawa T Nishino H Hasegawa M Ohta Y Iino Y Ichimura K Noda Y Interleukin-6 directly influences proliferation and invasion potential of head and neck cancer cells Eur Arch Otorhinolaryngol 2007 264 7 815 821 10.1007/s00405-007-0264-6 17310346

[B33] Hong DS Angelo LS Kurzrock R Interleukin-6 and its receptor in cancer: implications for Translational Therapeutics Cancer 2007 110 9 1911 1928 10.1002/cncr.22999 17849470

[B34] Ponzetto C Bardelli A Zhen Z Maina F dalla Zonca P Giordano S Graziani A Panayotou G Comoglio PM A multifunctional docking site mediates signaling and transformation by the hepatocyte growth factor/scatter factor receptor family Cell 1994 77 2 261 271 10.1016/0092-8674(94)90318-2 7513258

[B35] Ponzetto C Bardelli A Maina F Longati P Panayotou G Dhand R Waterfield MD Comoglio PM A novel recognition motif for phosphatidylinositol 3-kinase binding mediates its association with the hepatocyte growth factor/scatter factor receptor Mol Cell Biol 1993 13 8 4600 4608 7687741 10.1128/mcb.13.8.4600-4608.1993 PMC360084

[B36] Lee WJ Chen WK Wang CJ Lin WL Tseng TH Apigenin inhibits HGF-promoted invasive growth and metastasis involving blocking PI3K/Akt pathway and beta 4 integrin function in MDA-MB-231 breast cancer cells Toxicol Appl Pharmacol 2008 226 2 178 191 10.1016/j.taap.2007.09.013 17961621

[B37] Lee KH Choi EY Kim MK Hyun MS Jang BI Kim TN Kim SW Song SK Kim JH Kim JR Regulation of hepatocyte growth factor-mediated urokinase plasminogen activator secretion by MEK/ERK activation in human stomach cancer cell lines Exp Mol Med 2006 38 1 27 35 16520550 10.1038/emm.2006.4

[B38] Lee KH Kim JR Hepatocyte growth factor induced up-regulations of VEGF through Egr-1 in hepatocellular carcinoma cells Clin Exp Metastasis 2009 26 7 685 692 10.1007/s10585-009-9266-7 19526316

[B39] Zeng Q Chen S You Z Yang F Carey TE Saims D Wang CY Hepatocyte growth factor inhibits anoikis in head and neck squamous cell carcinoma cells by activation of ERK and Akt signaling independent of NFkappa B J Biol Chem 2002 277 28 25203 25208 10.1074/jbc.M201598200 11994287

[B40] Ma PC Tretiakova MS Nallasura V Jagadeeswaran R Husain AN Salgia R Downstream signalling and specific inhibition of c-MET/HGF pathway in small cell lung cancer: implications for tumour invasion Br J Cancer 2007 97 3 368 377 10.1038/sj.bjc.6603884 17667909 PMC2360323

[B41] Toschi L Janne PA Single-agent and combination therapeutic strategies to inhibit hepatocyte growth factor/MET signaling in cancer Clin Cancer Res 2008 14 19 5941 5946 10.1158/1078-0432.CCR-08-0071 18829470

